# Rare appendiceal escapades in childhood: the Grande experience!

**DOI:** 10.1093/jscr/rjab284

**Published:** 2021-07-14

**Authors:** Ashish Lal Shrestha, Govinda Adhikari, Gaurav Kattel, Manim Amatya

**Affiliations:** Department of Neonatal and Pediatric Surgery, Grande International Hospital, Kathmandu, Nepal; Department of Radiology, Grande International Hospital, Kathmandu, Nepal; Department of Pathology, Grande International Hospital, Kathmandu, Nepal; Department of Pathology, Grande International Hospital, Kathmandu, Nepal

## Abstract

Acute appendicitis in children is known to present in two broad forms: (1) uncomplicated and (2) complicated. Apart from this, a variety of atypical presentations can occur that may pose difficulty in diagnosis or treatment approach. We hereby present a series of such rare experiences namely appendiceal oxyuriasis, sub-hepatic appendicitis and appendiceal mucocele that were encountered and managed accordingly.

## INTRODUCTION

The life time risk of developing acute appendicitis (AA) throughout the age groups is 7% [1]. The inability to predict formidable complications like perforation and peritonitis influences early operative management [2]. It is important to have an accurate preoperative diagnosis to avoid a negative exploration, for example, mesenteric lymphadenitis; a very close mimic of AA may pose a significant diagnostic challenge in this regard [3]. Similarly, avoidance of operative treatment for an appendiceal oxyuriasis (AO) would be ideally recommended if diagnosed prospectively.

Also the modality of treatment is equally crucial for the best outcome. For instance, sudden right upper abdominal pain would normally be perceived as an upper abdominal pathology in most situations, until guided by sonological findings diagnosing sub-hepatic appendicitis (SHA), which dictates a modification in the surgical approach. Likewise, an appendiceal mucocele (AM) on preoperative sonology would be dealt with open approach to avoid peritoneal contamination when chances of malignancy are predictablyhigh.

## CASE SERIES

### Case 1

A 10-year-old boy presented to the emergency room with 2 days of abdominal pain initially over the peri-umbilical region and later localizing to right iliac fossa (RIF) with an episode of non-bilious vomiting. Physical examination revealed low-grade temperature (100°F) and tenderness with guarding overRIF.

Hematological tests showed polymorph nuclear leukocytosis with left shift without eosinophilia. Biochemical tests and urinalyses were normal. Abdominal radiographs were unremarkable. Ultrasonogram (USG) abdomen could not visualize the appendix, but reported significant probe tenderness inRIF.

With clinical impression of AA, he underwent diagnostic laparoscopy under general anesthesia.

**Figure 1:**
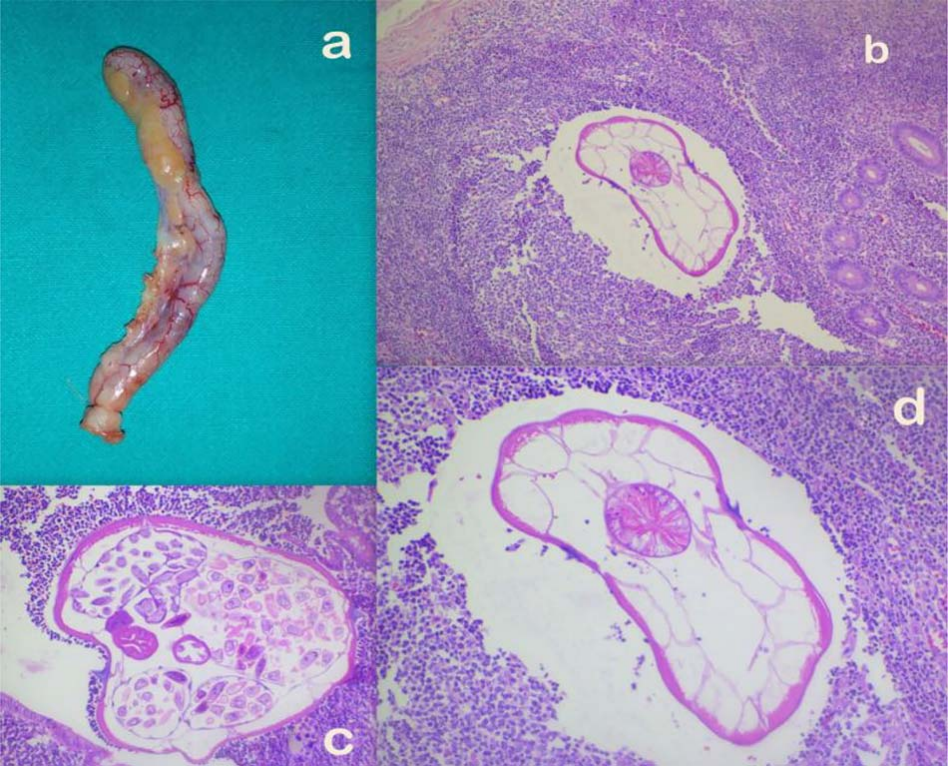
(**a**) Appendectomy specimen. (**b**–**d**) Microscopic appearance of AO at ×10 (b), ×20 (c) and ×40 (d) magnifications, respectively, showing mild congestion and luminal parasite.

Intraoperatively, a retro-cecally located appendix was found that was mildly inflamed with surface congestion more towards the tip (with visible leash of prominent vessels) and a healthy base as shown in Fig. 1a.

There was no perforation, peritoneal reactive fluid or pus and no omental reaction. Aware of his clinical presentation, a possibility of other pathologies like Meckel diverticulum, mesenteric lymphadenitis and any other small bowel lesions were thought of. On walking the bowel beyond 2 feet proximal to Ileo-Caecal Junction (ICJ), no other obvious lesions were found. Laparoscopic appendectomy was performed. Specimen was retrieved in a plastic bag in an attempt to avoid contamination.

Histopathologically, gross examination confirmed the operative findings showing a 5.5 × 0.7 cm appendix with venous congestion over the serosal surface. Microscopically, the section showed mild congestion and a luminal parasite with features compatible with *Enterobius vermicularis* as shown in Fig. 1b–d. The specimen was reported to be an AO. He had an uneventful postoperative period and was discharged the next day. At follow up a week later, he had recoveredwell.

The histopathological finding came as a diagnostic surprise, since historically he never complained of appendiceal colic (AC), anal pruritus or worm defecation in the past. Moreover, eosinophilia was also not observed in the peripheral smear. Attributing his symptom complex to manifestation of AO, he was treated with a single dose of albendazole to be repeated after 2 weeks. His family members were also advised a similar treatment course. At 1 year follow up, he remained symptomfree.

### Case 2

**Figure 2:**
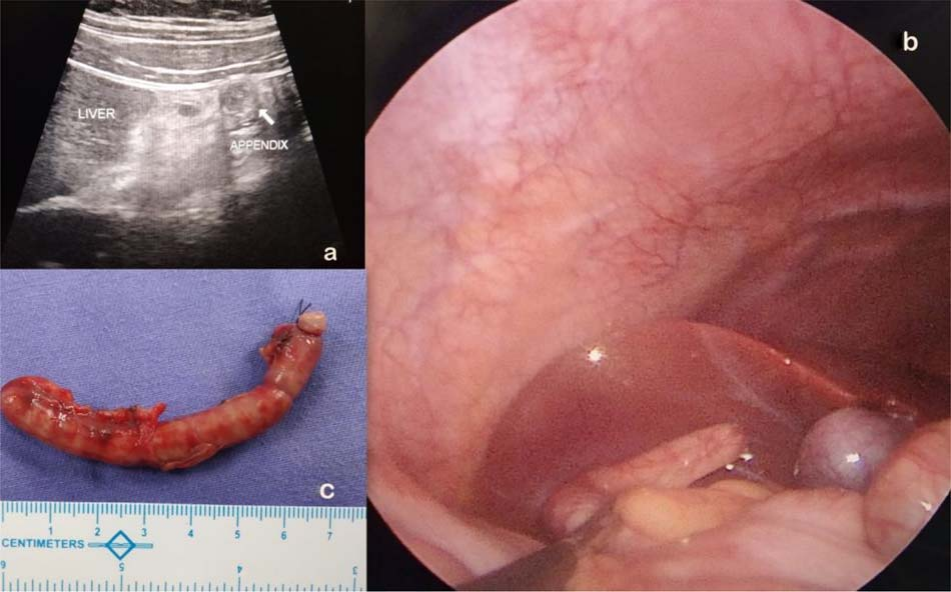
(**a**) SHA diagnosed and located on abdominal ultrasound. (**b**) Laparoscopic visualization of SHA. (**c**) Appendectomy specimen of theSHA.

A 10-year-old boy presented with history of peri-umbilical pain later migrating to right upper abdomen for 3 days and associated three episodes of non-bilious vomiting. He did not have fever, nausea or anorexia. He was moving bowel and bladder normally. Physical examination revealed tachycardia (110 beats per minute) and tenderness over entire right upper quadrant associated with rebound tenderness. Hematological tests were unremarkable. Biochemical tests were normal. Abdominal radiographs were unremarkable. USG abdomen showed a blind ending tubular, non-compressible, non-peristaltic structure measuring 9.5 mm, suggesting an enlarged appendix with its tip located in the sub-hepatic region as shown in Fig. 2a.

With clinical impression of SHA, he underwent an emergency diagnostic laparoscopy under general anesthesia. Intraoperatively, findings were confirmed, an inflamed turgid appendix with its tip just beside the gall bladder in the sub-hepatic region was noted with a healthy base as shown in Fig. 2b.

Laparoscopic appendectomy was performed as shown in Fig. 2c.

Histopathologically, gross findings included an 8 cm long appendix with cut section showing lumen filled with fecal material. Microscopically, the section showed suppuration, edema and congestion along with peri-appendiceal inflammation and focal areas of destruction of the muscular layer suggestive of gangrenousAA.

He had an uneventful postoperative period and was discharged the next day. At follow up a week later and 1 year later, he was doingwell.

### Case 3

**Figure 3:**
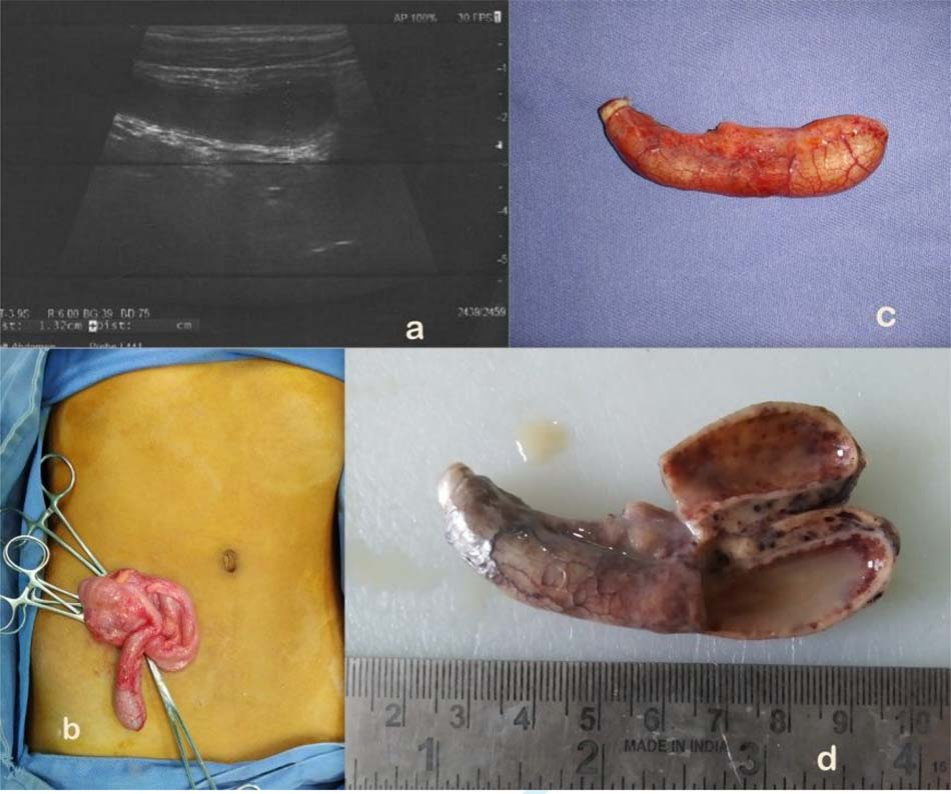
(**a**) AM as seen on abdominal ultrasound. (**b**) On table findings of AM at open appendectomy. (**c**) AM appendectomy specimen. (**d**) Gross pathology showing appendiceal lumen showing purulent material, thinned out wall and no obvious mass lesion.

A 13-year-old boy presented with sudden onset RIF pain for a day associated with vomiting, nausea and anorexia without fever. He was moving bowel and bladder normally. Physical examination revealed guarding and rebound tenderness over RIF. Hematological tests showed polymorph nuclear leukocytosis. Biochemical tests and urinalyses were normal. Abdominal radiographs were unremarkable. USG abdomen showed a blind ending tubular, fluid-filled, non- compressible, non-peristaltic structure in RIF measuring 13 mm, suggesting an enlarged appendix with mucocoele formation as shown in Fig. 3a.

In view of known associations of mucocoele with malignancy, he underwent open appendectomy as shown in Fig. 3b and c. No visible or palpable mass lesion was found intraoperatively. Histopathologically, gross findings included an 8 cm long appendix with cut section showing lumen filled with purulent material and thinning of wall (2 mm) without an obvious mass lesion as shown in Fig. 3d. Microscopically, the section showed suppuration, edema and congestion along with mucosal erosions. The lumen was filled with neutrophils and fibrinoid material. Peri-appendiceal inflammation was noted along with focal areas of destruction of the muscular layer suggestive of gangrenousAA.

He had an uneventful postoperative period and was discharged the next day. At follow up a week later and 1 year later, he was doingwell.

## DISCUSSION

Ever since the theory of luminal obstruction and consequent infection was suggested by Zwalenberg in1905, numerous etiologies have been implicated in the causation and clinico-pathological manifestation of AA [4–6]. AO, a lesser known cause, is said to be associated in 0.2–41.8% of AA diagnosed worldwide [7]. Enterobius (pin worm) is the causative agent for AO and the commonest gut parasite affecting 4–28% of children between 5 and 14 years [5].

Although known to cause a wide variety of gut problems like ileocolitis, perianal abscesses, enterocutaneous fistulae and mesenteric abscesses, AO is still not a common cause of AA [8–12]. It is not easy to establish its causal relationship with a truly invasive inflammation even when a theoretical possibility of AC does exist [1, 10, 11, 13]. Operative treatment in such patients is considered arguable, although in retrospect.

However, the diagnosis cannot possibly be confirmed until the histopathological analysis. Therefore, in patients with intraoperative findings that are incongruent with the clinical picture, the suspicion of this lesser known pathology should be borne in mind. This group of children could possibly benefit with observations and serial assessments to avoid operation. Meanwhile, fecal sampling and night time cellophane tape application over the perineum to detect the parasite as well as empirical antihelminthic therapy could be instituted [14]. A similar scenario was encountered with Case 1, with typical symptom complex of AA, wherein appendectomy could not be avoided.

Likewise, the appendicular location could impose a diagnostic challenge when presenting with acute symptoms of inflammation [15]. The appendix is commonly located in the retrocecal position in 65% cases and less commonly in the pelvis, subcecal, pre- and post-ileal locations in the decreasing order of frequency. Rarer positions include sub-hepatic, lateral pouch, mesocoeliac, left-sided and intraherniary, with reported incidence of SHA being 0.08% [16]. This was first reported by King in 1955 [15, 17].

Owing to its location, AA in sub-hepatic position mimics hepatobiliary or gastro duodenal pathologies, clinically leading to a delayed diagnosis and complications like suppuration, perforation, intra-abdominal abscess and sepsis [18]. With a few cases described in children, a strong index of clinical suspicion is imperative to counter aforementioned complications. Such was the experience with Case 2.

With symptom complex of AA and all the findings located over the right upper abdomen, it could have been easily mistaken for an upper abdominal pathology. Even though a cross-sectional abdominal imaging like computed tomography scan has been favored by a few studies, in our patient, an abdominal ultrasound was adequate for diagnosis [15]. Laparoscopic approach was used to localize the infection and subsequent appendectomy could be achieved using a standard three port technique. This was of dual benefit as it avoided a conventional Lanz incision that was unsuitable to locate a cranially situated appendix and also a right sub-costal muscle cutting incision that is known for its morbidity, besides the usual advantages of laparoscopy [19].

Lastly, the surgical approach to AA also depends on preoperative predictability of sinister findings as with an appendicular mucocele, which was diagnosed on ultrasound in Case 3.

Mucocele of the appendix is an exceedingly rare presentation of AA in children with scarce descriptions and sporadic reports [20]. It implies an appendix that is cystic and dilated with mucinous material as a result of an obstructive lesion. This could be a fecalith, endometriosis, extrinsic compression, inflammation or even a neoplasm. Appendicular malignancies, with an incidence of <0.5%, are a rare occurrence in children with predominant ones being neuro-endocrine variety [21].

With a clinical presentation often mimicking AA, mucinous neoplasms of appendix are difficult to distinguish clinically. Since, tactile examination to confirm tumor in the adjacent large bowel is desirable, an open approach was favored in Case 3 to deal with this potential pathology. However, with normal findings, the procedure could be concluded with a simple appendectomy.

## CONCLUSION

1. When on table findings are incongruent with the clinical picture, rarer cause of AA like AO needs to be considered.
2. When symptom complex of AA does not follow a clinico-anatomical correlation, a developmental malformation like abnormal appendico-cecal location must be suspected.
3. When symptom complex of AA is accompanied by an uncommon radiological finding, the surgical approach should be guided by the foreseeable intraoperative findings.
